# Debate: Emergency mental health presentations of young people during the COVID‐19 lockdown

**DOI:** 10.1111/camh.12411

**Published:** 2020-08-18

**Authors:** Dennis Ougrin

**Affiliations:** ^1^ Department of Child & Adolescent Psychiatry King's College London London UK

Suicide is the second leading cause of death in young people in most developed countries, exceeded only by accidents. Self‐harm is one of the strongest known predictors of suicide (Hawton et al., 2012). Both suicide and self‐harm in young people have been rising in the UK, and, in recent years, social media has been playing a prominent role in the way young people communicate about self‐harm (Sedgwick et al., 2019). Accident and Emergency (A&E) presentations with self‐harm have seen a particularly sharp increase before the COVID‐19 lockdown, and the number of young people seeking help for self‐harm has also increased in primary care (Morgan et al., 2017). There has also been an increase in the number of young people reporting self‐harm in surveys of the general population, especially older girls. These increases in self‐harm have been paralleled by increases in suicide in older teenagers (15–19‐year‐olds), rising from 4.1 to 6.7/100,000 between 2010 and 2018 (ONS, 2019).

Although at least three quarters of young people with self‐harm do not access mental health services (Ystgaard et al., 2009), severe self‐harm is a common cause of inpatient admissions. There has been a significant increase in the number of psychiatric inpatient admissions in England between 2008 and 2018 which then plateaued and started to decrease in 2019 (NHSEngland, 2019). The reasons for this recent reduction could be complex; however, a rapid expansion of intensive community care services may have been a deciding factor. Lengthy inpatient admissions for young people with self‐harm, although sometimes needed, have been linked with an increase in self‐harm (Ougrin et al., 2018, 2014; Reavey et al., 2017) although not in the risk of A&E presentations with self‐harm (Ougrin et al., 2020).

The outbreak of COVID‐19 in the UK and the associated quarantine (lockdown) measures are likely to have had a substantial influence on young people’s mental health. Previous epidemics have been associated with an increase in emotional disorders and neuropsychiatric disorders although the impact of these epidemics has been poorly studied outside of the most affected nations. The pandemic started in China, in December 2019. As of the 30th of June 2020, more than 10 M people have contracted the disease and more than 500,000 people have died.

To assess the impact of the lockdown measures on young people’s mental health, we used the National Commissioning Data Repository (NCDR), an England‐wide dashboard providing the most up‐to‐date data on a number of health services, including child and adolescent mental health services (Ougrin, 2020). NCDR contains information on temporal changes in inpatient psychiatric admissions of children and adolescents in England. We then used NHS Digital data on the total number of A&E presentations in March and April 2020 compared with March and April 2019. Using the data from NCDR and NHS Digital, we sought to establish (a) changes in the number of A&E presentations after the establishment of the quarantine measures (March and April 2020) and in comparison with the March–April period of the previous year (b) changes in the number of inpatient admissions for psychiatric emergencies during the same periods.

We then used local electronic patient data to investigate the A&E presentations in detail, comparing the data on the A&E presentations of young people with psychiatric symptoms between March and April 2020 and March and April 2019. We then compared the UK findings about the A&E presentations with the data from several other countries. We hypothesised that the total number of A&E presentations will be lower in March and April 2020 versus March and April 2019. For a detailed account of these studies, please see (Ougrin, 2020).

Children and adolescents aged 0–17 years (inclusive) who either presented to A&E or were admitted to inpatient care with mental health disorders were included in this cross‐sectional study. Self‐harm was defined using the UK National Institute for Health and Care Excellence clinical guidelines as 'any act of self‐poisoning or self‐injury, irrespective of the underlying intent (NICE), thus incorporating both nonsuicidal self‐injury, suicide attempts nonsuicidal self‐poisoning and self‐harm with unclear or mixed intent'.

Using the NHS Digital database, the total number of A&E presentations in England in April 2020 was 917,000, a decrease of 56.6% on the same month last year. These are the lowest number of attendances reported since this collection began and are likely to be a result of the COVID‐19 response. There was a smaller but significant reduction in March 2020 compared to March 2019.

Using the NCDR database, the total number of psychiatric inpatient admissions for young people in England in March 2020 was 217 (384 in 2019, 359 in 2018 and 414 in 2017 and 2016), the lowest number of admissions in any month since records began. The number of admissions was even lower in April 2020 (244 vs. 398 in 2019, 391 in 2018 and 366 in 2017 and 368 in 2016).

There was a substantial decrease in the number of A&E presentations in young people for psychiatric reasons and a substantial decrease of A&E presentations for self‐harm in most countries studied (Ougrin, 2020).

The lower incidence of A&E presentations with psychiatric symptoms, including self‐harm among this age group, not compensated for by an increase in the number of inpatient admissions is significant. It is potentially due to the quarantine measures. Of importance, many children and adolescents stopped going to school in March and April 2020 and the pressure to perform during the examination periods was reduced. In addition, many young people are likely to have stayed at home more often, interacting with their parents and feeling part of a family more than before. Many young people may have had fewer opportunities to engage in face‐to‐face encounters associated with increased risk of self‐harm and worsening of psychiatric disorders, such as face‐to‐face bullying, using drugs and alcohol and engaging in risky behaviour. Although some evidence indicates that common mental health disorders, especially emotional disorders are becoming more common during the periods of epidemics and that some young people might be at greater risk of violence and abuse at home, the numbers affected might be relatively low. Exposure to digital media and its potential impact on children and adolescents’ mental health is the centre of continued media debate. Such technologies can be helpful and facilitate access to care and support, but there is also a suggestion that extreme 'connectedness' could have detrimental effects. The most plausible explanation for our findings could be that the marked decrease in incidence observed reflects both a true decline in incidence together with less frequent help‐seeking behaviour. This could mean that a significant number of young people with severe psychiatric symptoms and self‐harm are not seeking help.

In summary, the studies undertaken by our group provide a unique hospital care perspective on self‐harm and inpatient admissions among children and adolescents during the COVID‐19 lockdown. It is not clear how long the decrease in A&E presentations and inpatient admissions is likely to last and if a compensatory increase following the lockdown will ensue.

The findings have major implications for service planning, especially if there is a second wave of COVID‐19 or a future pandemic or a lockdown for any other public health emergency. Services should be reconfigured in such a way that A&E staff are prepared for lower numbers of presentations and redeployment of staff from hospital‐based care services to home treatment and online services should be prioritised.

## Ethical information

The work was approved by South London and Maudsley NHS Foundation Trust service evaluation and clinical audit committee 11/06/2020.
